# Polish Experiences of Pain Treatment by Paramedics in relation to Good Practices of Pain Treatment: A Register-Based Study

**DOI:** 10.1155/2022/3677688

**Published:** 2022-04-07

**Authors:** Mariusz Koral, Jakub Szyller

**Affiliations:** ^1^Medical Simulation Center, Faculty of Medicine, Wroclaw Medical University, Wroclaw, Poland; ^2^Division of Clinical Chemistry and Laboratory Hematology, Department of Medical Laboratory Diagnostics, Faculty of Pharmacy, Wroclaw Medical University, Wroclaw, Poland

## Abstract

**Background:**

Pain is frequently encountered in the prehospital setting. The treatment of pain is one of the priority activities for paramedics.

**Methods:**

1048576 patients under the care of EMS over a 7-month period were included in this study. Data included using pain scale and pain score, the initial diagnosis, analgesic treatment and the given drug obtained from the Ambulance Emergency Procedure Cards.

**Results:**

The complete absence of pain was detected in 43.9% (95% CI 43.8–44.0) of patients, mild pain in 17.0% (95% CI 16.9–17.1), moderate pain in 11.6% (95% CI 11.5–11.7), and severe pain in 4.9% (95% CI 4.9–4.9) of cases. In 22.5% (95% CI 22.4–22.6), no pain rating scale was used. 98.0% (95% CI 97.9–98.1) of patients with mild, 96.1% (95% CI 96.0–96.2) with moderate, and 94.0% (95% CI 93.9–94.1) with severe pain did not receive any pain medication. The most commonly used drugs in all groups were metamizole and ketoprofen. The greatest analgesic effect was observed in the group of patients with severe pain. Pain decreased by an average of 3.2 NRS points in the drug-free group and 3.1 NRS points in the treated group. The most commonly documented diagnoses in all groups of patients were signs and diseases not classified in other groups (ICD-10 R00-R99), injuries and consequences of external causes (ICD-10 S00-T98), and diseases of the circulatory system (ICD-10 I00–I99).

**Conclusions:**

Paramedics may use a variety of medications but use them rarely. Adding metamizole to the list of medications used by paramedics has made it one of the most commonly used drugs. Regardless of whether the drug was used or not, the analgesic effect was similar.

## 1. Introduction

Pain is a subjective complex phenomenon caused by illnesses or injuries. The extension of paramedic's competences in legal regulations in Poland in the beginning of 2016 provoked broad discussion among clinical emergency doctors, researchers in the area of pain, and paramedics themselves. One of the main causes of doubt was the possibility of self-administration of analgesic drugs (without a doctor's order but at the discretion of the Emergency Medical Service (EMS) crew), such as ibuprofen, metamizole, paracetamol, and fentanyl. Paramedics could earlier use only acetylsalicylic acid (ASA), ketoprofen, and morphine. The National Medical Rescue System in Poland is based mainly on the so-called primary ambulance emergency teams without a medical doctor that consist of two or three paramedics or emergency nurses. Paramedics ensure the professional care and implementation of rescue procedures at the Advanced Life Support (ALS) level. They can now independently administer 47 drugs, including analgesic agents. The following analgesic drugs are nonopioids: ibuprofen (tablets), ketoprofen (tablets and intravenous), sodium metamizole (intravenous), paracetamol/acetaminophen (tablets, intravenous, and suppositories), and acetylsalicylic acid (tablets), as well as opioids: morphine and fentanyl (both intravenous). Finally, they are entitled to administer drotaverine and papaverine which (together with ASA) were not taken into consideration in this study. Paramedics most often use the numeric rating scale (NRS) or similar (visual analogue scale (VAS) or Faces Pain Scale (FPS), also in the range of 0–10 points) to assess pain. In most cases, the assessment is made twice—before and after the treatment procedures (taking into account the pharmacokinetics of various drugs and the condition of the patient). Only some rare reports on prehospital pain management by paramedics in Poland were available and they focused on regional research material and their results were not optimistic [1–6]. Limited literature data and daily clinical practice show that paramedics are usually able to assess pain properly and choose the right analgesic agent according to the patient status but they do it too rarely [4]. Before 2014, paramedics were unable to effectively treat nontraumatic pain and fever, thus addition of new drugs significantly improved the working comfort of EMS which is confirmed by the analysis of Kiszka et al., where the authors noticed that after the extension of paramedic skills, they most frequently used metamizole and paracetamol, respectively in 8.0% and 3.1% of patients who were given analgesic drugs [3]. The majority of pain treatment reports concerns trauma patients. Interestingly, Zdunczyk et al. showed that over half of traumatic patients was not treated for pain in the prehospital care and those who were given such drugs received nonsteroid anti-inflammatory drugs (NSAIDs) more often than opioids [7]. Our retrospective analysis from 2013 on the sample of over 20000 Ambulance Emergency Procedure Cards showed that paramedics used opioids in the pain treatment only in 0.7% of all procedures [5]. In the study conducted in the north-western Poland after 2016, only 14.5% of such patients received analgesic drugs (19.7% in multiple trauma cases) [2]. In 2016, a document “Good Practices of Pain Treatment by Emergency Medical Services” was developed and four different versions were created: (i) Primary Ambulance Emergency Teams, for adults, (ii) Primary Ambulance Emergency Teams, for children, (iii) Specialized Ambulance Emergency Teams and Helicopter Emergency Medical Services (HEMS), for adults, and (iv) Specialized Ambulance Emergency Teams and HEMS, for children. The detailed description is shown in Table 1. So far, no detailed analysis of using of new analgesic drugs by paramedics or of practical implementation of issued recommendation has been carried out. There are no data describing the clinical situations in which the drugs were administered and assessing the effectiveness of analgesic treatment. The aim of the study was to determine how often paramedics use analgesic drugs, in what situations and what analgesic effect they achieve. Our study provides an answer to this missing information. Special attention has been paid to assessing pain levels and to multimodal analgesia as the optimal treatment for acute and chronic pain in patients in prehospital care. We analyzed the situations in which specific drugs were used, the severity of pain, and the analgesic effect expressed in points of the 0–10 scale.

## 2. Materials and Methods

### 2.1. Methods

This research is a register-based observational study of patients with acute pain transported by emergency medical services (EMSs) to hospital or left at the call place in Poland over a 7-month period from January 1, 2020 to July 31, 2020 (1048576 patients). The research material consisted of data gathered from Ambulance Emergency Procedure Cards collected in the System of Management Assistance for the National Medical Rescue Service. The authors applied to the Ministry of Health for access to data concerning several actions and procedures implemented by EMS in the whole area of Poland. It was information about using pain scale, patient assessment with the NRS and the score (first and repeated, when applicable), the initial diagnosis made by paramedics (according to ICD-10 classification code and description), analgesic treatment and the given drug and, finally, using nonpharmacological techniques (e.g., immobilization). The latter information was not complete, so it was not considered in the final analysis. The material received from the National Monitoring Center of Medical Rescue System did not include any demographic data such as age and gender of the patients, either due to very strict legal regulation about personal data protection. The records with at least one application of numerical pain assessment scale were selected first. They were subsequently divided into subgroups according to the document “Good Practices of Pain Treatment by Emergency Medical Services,” such as: mild pain (1–4 pts.), moderate pain (5–7 pts.), and severe/extreme pain (8–10 pts.). In each subgroup, we analyzed applied analgesic drugs, indications for their use, and also diagnoses in which no pharmacological agents were given. The groups were also created in which the NRS score was zero. Moreover, the authors analyzed the frequency of each drug application depending on pain intensity and initial diagnosis according to ICD-10 classification. The key to the list of diagnoses compatible with ICD-10 classification is shown in Additional file 1.

### 2.2. Data Analysis

Statistical analysis was performed using GraphPad Prism ver. 8.4.0, GraphPad Software, San Diego, California USA. Categorical data were reported as numbers/percentages (%) with 95% confidence intervals (CI). The means were reported as mean ± standard deviation (SD). Due to extremely large study material, no standard statistical tests were used in order to compare the pain in different groups of patients, because they might show statistical relevance with negligible change in the NRS, so their clinical importance would be minimal. In such cases, the data were shown in a descriptive and illustrative manner.

## 3. Results and Discussion

### 3.1. General Results

In total, 1048576 interventions of primary EMS were analyzed. The complete absence of pain (0 pts. in the NRS) was detected in 43.9% (95% CI 43.8–44.0, *n* = 460569) of patients, mild pain in 17.0% (95% CI 16.9–17.1, *n* = 178723), moderate pain in 11.6% (95% CI 11.5–11.7, *n* = 121214), and severe/unbearable pain in 4.9% (95% CI 4.9–4.9, *n* = 51617) of cases. In 22.5% (95% CI 22.4–22.6, *n* = 236433) of people, no pain rating scale was used. In less than 0.01% (95% CI 0–0.01, *n* = 20) of patients, it was not possible to unequivocally determine the use of the NRS or whether the assessment was repeated. Analgesic drugs were used in 1.0% (95% CI 1.0–1.0, *n* = 10671) of all analyzed interventions of EMS.

### 3.2. Mild Pain: Without Pharmacotherapy

**Figure 1:**
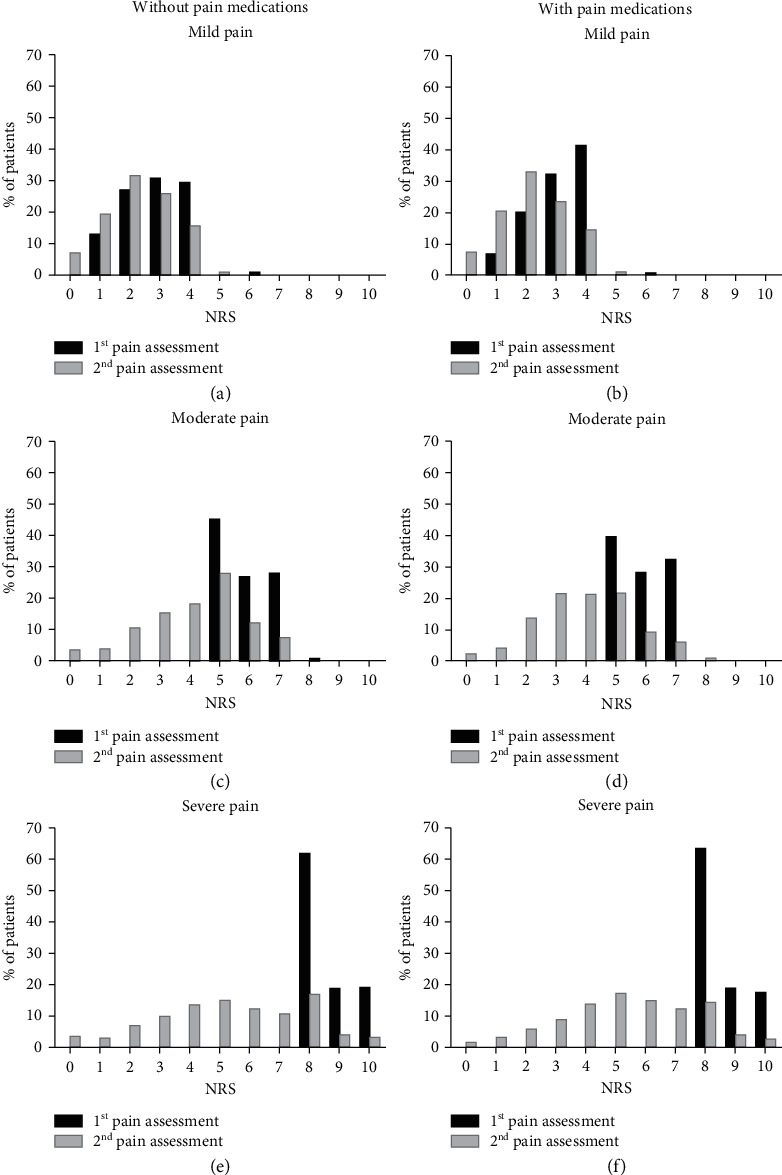
Pain in the group with and without medications. Patients with mild (a), moderate (c), and severe pain (e) at the first and second paramedics' pain assessment who have not received any pain medication, and patients with mild (b), moderate (d), and severe (f) pain at the first and second pain assessment who have received nonopioid or opioid pain medication.

As many as 98.0% (95% CI 97.9–98.1, *n* = 175284) of patients with mild pain did not receive any pain medication. Patients with 3 pts. in the first and 2 pts. in the second assessment were the largest group (Figure 1(a)). The second examination was performed in 93.7% (95% CI 93.5–93.9) of patients. Pain disappeared completely in 6.9% of patients. Detailed statistical data are shown in Additional file 2. The most frequent diagnosis (like in each other analyzed groups) were signs and diseases not classified in other groups (ICD-10 R00-R99, 39.9%, 95% CI 39.7–40.1) and injuries and consequences of external causes (ICD-10 S00-T98, 21.6%, 95% CI 21.4–21.8). Diseases of the circulatory system (ICD-10 I00–I99) were reported in 13.9% (95% CI 13.7–14.1) of patients (see Additional file 3).

### 3.3. Mild Pain: Use of Analgesic Agents

**Figure 2:**
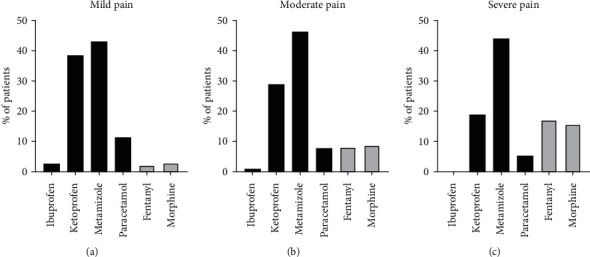
The frequency of use of analgesics in the group with mild (a), moderate (b), and severe (c) pain. Black colour indicates nonopioid drugs, and gray indicates opioids.

The pain intensity in the pharmacotherapy group ranged from 1 to 4 pts. during the first assessment and the majority of patients (41.3%) graded their pain as 4 pts. In the second examination, the most frequent result (33.0%) was only 2 pts (Figure 1(b)). Analgesic drugs were given to only 1.9% (95% CI 1.8–2.0, *n* = 3438) of patients with mild pain. The medications were as follows: ibuprofen, 2.4% (95% CI 1.9–2.9, *n* = 81), ketoprofen, 38.4% (95% CI 36.8–40.0, *n* = 1319), metamizole, 43.0% (95% CI 41.3–44.7, *n* = 1480), paracetamol, 11.3% (95% CI 10.2–12.4, *n* = 390), fentanyl, 2.1% (95% CI 0.8–1.4, *n* = 73), and morphine, 2.8% (95% CI 2.2–3.4, *n* = 95) (Figure 2(a)). The second pain assessment was made in ≥95.0% of cases. Medications were most frequently used in patients with 3- and 4-pt pain score. Regarding nonopioid drugs, metamizole was commonly used (42.6%) in patients with 4 pts. NRS, ibuprofen, and ketoprofen were used also in the most severe pain in this group (4 pts., same in 40.7% of cases), and considering opioid agents, morphine and fentanyl, at 4 pts. The detailed correlation of drug application with pain intensity is shown in Additional file 4. All analyzed analgesics were applied most frequently in signs and diseases not classified in other groups (ICD-10 R00-R99, mainly paracetamol in 56.7% of patients with this diagnosis) and in injuries and consequences of external causes (ICD-10 S00-T98, mainly morphine in 68.1%). The considerable number of these patients suffered also from diseases of the circulatory system as well as diseases of the musculoskeletal system and connective tissue. Additional file 5 shows the frequency of each drug application depending on the diagnosis.

### 3.4. Moderate Pain: Without Pharmacotherapy

As many as 96.1% (95% CI 96.0–96.2, *n* = 116433) of patients with moderate pain did not receive any analgesic treatment. The pain score range in this group was 5–7 pts. and the most common score was 5 pts. in 45.3% patients. The second pain assessment was made in 92.2% patients (Figure 1(c), Additional file 6), and the number of patients with the most severe pain (7 pts.) decreased during the second examination (despite not using analgesics, from 27.5% to only 7.5%). The most common were signs and diseases not classified in other groups (ICD-10 R00-R99, 46.9%, 95% CI 46.7–47.1) and also injuries and consequences of external causes (ICD-10 S00-T98, 23.8%, 95% CI 23.6–24.0). The detailed diagnoses consistent with the ICD-10 classification are shown in Additional file 3.

### 3.5. Moderate Pain: Use of Analgesic Agents

In the group of patients with moderate pain, medications were used in 3.9% (95% CI 3.4–4.4, *n* = 4781) of cases. The first examination showed that the majority of the patients rated the pain as 5 pts. (39.6%). In the second assessment, the score ranged from 0 to 10 (Figure 1(d), Additional file 6). The patients were given: ibuprofen, 0.3% (95% CI 0.1–0.5, *n* = 14), ketoprofen, 28.9% (95% CI 27.6–30.2, *n* = 1381), metamizole, 4.3% (95% CI 44.9–47.7, *n* = 2214), paracetamol, 7.7% (95% CI 6.9–8.5, *n* = 366), fentanyl, 8.1% (95% CI 7.3–8.9, *n* = 389), and morphine, 8.7% (95% CI 7.9–9.5, *n* = 417) of cases (Figure 2(b)). The second assessment of pain was performed in ≥95.0% patients in each of the used drugs. The medications were most commonly used in patients with pain on 6 and 7 pts. Concerning nonopioid drugs, metamizole and ketoprofen were applied most frequently, especially in patients with 5 pts. Paracetamol and ibuprofen were also mainly applied in patients with 5 pts. In the most severe pain (7 pts.) in the whole group of patients with moderate pain, fentanyl was mainly used in the people with the score of 7 (47.0%). The detailed distribution of drug application depending on the pain intensity is shown in Additional file 7. All analyzed medications were given most often in injuries and consequences of external causes (ICD-10 S00-T98, mainly morphine, 62.5%) and also in signs and diseases not classified in other groups (ICD-10 R00-R99, mainly metamizole, 58.4%). Additional file 8 presents the detailed frequency of each drug application according to the diagnosis.

### 3.6. Severe/extreme Pain: Without Pharmacotherapy

As many as 94.0% (95% CI 93.9–94.1, *n* = 49165) of patients with severe pain were not given any drugs. The largest group of people (62.0%) had the 8-pt pain score (Figure 1(e)). The second examination was made in 96.7% (95% CI 96.5–96.9, *n* = 47466) of patients. 8-pt pain decreased from 62.0% to 17.3% of patients and in case of 10 pts. decreased from 19.2% to 3.3%. The details are given in Additional file 9, and the ICD-10-based diagnoses in these patients are shown in Additional file 3. The most frequent disorders were signs and diseases not classified in other groups (ICD-10 R00-R99, 43.7%, CI 9%% 43.5–43.9) and also injuries and consequences of external causes (ICD-10 S00-T98, 25.2%, 95% CI 25.0–25.4).

### 3.7. Severe/Extreme Pain: Use of Analgesic Agents

Analgesic drugs were administered in only 4.8% (95% CI 4.6–5.0, *n* = 2452) of patients with severe/extreme pain. The pain severity in the first examination ranged from 8 to 10 pts. In the repeated assessment, the pain score ranged from 0 to 10 pts., with the mean of 5.4 ± 2.3 points which shows a considerable pain reduction (see Additional file 9). The patients were given: ketoprofen, 18.5% (95% CI 17.0–20.0, *n* = 453), metamizole, 43.9% (95% CI 41.9–45.9, *n* = 1076), paracetamol, 5.2% (95% CI 4.3–6.1, *n* = 128), fentanyl, 17.0% (95% CI 15.5–18.5, *n* = 416), and morphine, 15.5% (95% CI 14.1–16.9, *n* = 379). The second examination was made in at least 96.1% of patients. Additional file 10 shows detailed correlation between the used drugs and pain severity. All drugs were most commonly given to patients with the 8-pt pain score. Opioid medications (mainly morphine) were administered more often in patients with 8 pts. Patients with the most severe pain (10 pts.) received fentanyl or morphine (not including multimodal analgesia). Additional file 11 shows the detailed frequency of each drug administration depending on the diagnosis. The most common diagnosis were signs and diseases not classified in other groups (ICD-10 R00-R99, 46.9%, 95% CI 46.7–47.1) and also injuries and consequences of external causes (ICD-10 S00-T98, 23.8%, 95% CI 23.6–24.0).

### 3.8. Multimodal Analgesia

As many as 1.5% (95% CI 1.1–1.9, *n* = 53) of patients with mild pain, 2.6% (95% CI 1.1–4.1, *n* = 126) with moderate pain, and 8.4% (95% CI 8.2–8.6, *n* = 205) with severe pain were given more than one analgesic drug. Detailed data are shown in Table 2.

### 3.9. Mild Pain

**Figure 3:**
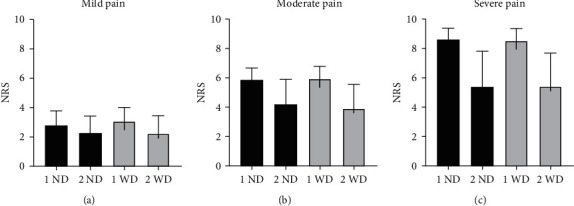
Pain severity in patients with mild (a), moderate (b), and severe (c) pain who did not receive (no-drugs group, ND) analgesic drugs (in the first and second assessments: 1ND, 2ND) and patients who received the drug (with-drugs group, WD; in the first and second assessments: 1WD, 2WD respectively).

In patients with mild pain (Figure 3(a)), who did not receive any medications, the intensity of pain in the first assessment was 2.8 ± 1.0 pts. and in the second 2.3 ± 1.2 pts. (Δ = 0.5 pts.). The patients who were given analgesic drugs scored the mean of 3.1 ± 0.9 pts. in the pain scale during the first examination and 2.2 ± 1.2 pts. in the second, Δ= 0.9 pts. These differences were not clinically significant because we originally assumed that only changes in the pain score above 2 pts. are relevant. Comparing the patients with and without analgesia, the pain severity was 3.1 ± 0.9 pts. and 2.8 ± 1.0 in the first assessment, Δ = 0.3 pts, and 2.3 ± 1.2 and 2.2 ± 1.2 pts. in the second assessment, Δ = 0.1 pts.

### 3.10. Moderate Pain

In patients with moderate pain (Figure 3(b)), who were not given pain medications, the pain severity in the first assessment was greater than in the second and amounted 5.8 ± 0.8 pts. and 4.2 ± 1.7 pts., respectively (Δ = 1.6 pts.). The similar difference was noted in patients from the pharmacotherapy group. During the first assessment, the mean score was 5.9 ± 0.8 pts. and 3.9 ± 1.6., Δ= 2.0 pts. In this group of patients, much greater differences were noted than in the mild pain group which was certainly translated to the patient status. No relevant differences in pain intensity were seen between people with and without pharmacotherapy. These were respectively 5.8 ± 0.8 and 5.9 ± 0.8 pts., Δ = 0.1 pts in the first examination and in the repeated one 4.2 ± 1.7 and 3.9 ± 1.6 pts., Δ = 0.3 pts. Neither base nor final pain score differed between the groups with and without drug administration.

### 3.11. Severe/Extreme Pain

Regarding the group of patients with severe pain (Figure 3(c)), who did not receive any analgesic treatment, the pain intensity during the first examination was greater than in the repeated assessment: respectively, 8.6 ± 0.8 pts. and 5.4 ± 2.4 pts., Δ = 3.2 pts. The similar situation was detected in the pharmacotherapy group. In the initial assessment, the mean score amounted 8.5 ± 0.8 pts. and in the second one 5.4 ± 2.3, Δ = 3.1 pts. This difference was the greatest of all analyzed populations. However, no relevant pain intensity changes were reported in people with and without drug treatment. These were: 8.6 ± 0.8 and 8.5 ± 0.8 pts., Δ = 0.1 pts. in the first assessment and 5.4 ± 2.4 and 5.4 ± 2.3 pts., Δ= 0.0 pts in the repeated examination. No visible difference in pain intensity change was therefore detected between patients treated and not treated despite of sensation of the most severe, extreme pain.

## 4. Discussion

The treatment of pain at the prehospital care level is a major concern in many healthcare systems all over the world [8–11], being one of the priority activities for paramedics [12]. The first large-scale analysis in Poland concerning application of pain assessment showed how often and in what situations paramedics use pain scales, what medications and whether multimodal analgesia they use and with what effect. The electronic system of management assistance and digital medical documentation give the opportunity to use one of the three pain scales—NRS, VAS, and FPS. Different diagnostic tools can help assess the pain and create more opportunities (e.g., in pediatric patients) and greater probability of precise pain intensity assessment [13, 14]. Our results show that pain of any intensity was reported in 33.5% of patients which is consistent with the results of Friesgaard et al., where moderate or severe pain was present in about 28% of EMS patients [15]. In total, paramedics used pain assessment scales in 77.5% of cases which is a considerable increase compared to the previous reports. In the analysis made in 2014, the NRS was used only in 22.5% of patients with pain [16]. Such great increase of using pain assessment tools may result from the continuous education of paramedics, legal changes related to operation of the National Medical Rescue System, and indirectly by the changes in the Act on Patients' Rights. On the other hand, there is a big problem. Only 1% of all patients and approximately 3% of patients with any pain level were receiving one or more pain medications. According to authors' experience, introduction of pain assessment protocol did not improve the frequency of analgesics use. A similar phenomenon was described by Jaeger et al. The analysis of the newly introduced pain assessment protocol that was incorporated into the medical documentation in children residing in the Salt Lake City District concerning the frequency of analgesic drug application showed similar results as in the present study. An increase in documented cases of using pain assessment scales (from 25% to as much as 100%) was observed but it had no visible influence on the frequency of drug application [17]. The study of McLean et al. shows that the 30 minutes training of paramedics, consisted of introducing pain assessment scales and practical information about local methods of pain examination, resulted in 84% frequency of using pain assessment protocols by means of two scales—NRS and VAS [18]. Similar results were obtained in the comparative analysis of the influence of education of Polish 5-year medicine students with the classic and simulation methods on the efficiency of acute traumatic pain treatment [19]. It was confirmed that using the simulation methods during university education resulted in more effective use of pain intensity assessment scales [19]. Moreover, it should be stressed that professional behaviors of young paramedics are to the great degree formed by more experienced staff who do not teach good practices. However, there are no Polish studies on the frequency of pain medications use.

During their daily routine, paramedics had contact with patients reporting pain symptoms: mild (1–4 points) in 17% of cases, including 1.9% of patients who were given analgesic drugs; moderate pain (5–7 points) in 11.6% of cases in whom 3.9% obtained analgesics; and extreme pain (8–10 points) in 4.9% of patients, including only 4.8% with applied pain medications. Berben et al. carried out a study to assess the conduct of Dutch national EMS staff in terms of using analgesia protocols in trauma patients and noticed that local paramedics applied pain medications in 42% of cases in all pain intensity scores [20]. Data were from Australia where 315000 cases were analyzed, as many as 34.5% of patients reported pain of any level and 20.3% of them were given opioids [21]. In a large (more than 41000 participants) study of Danish prehospital care system, 27.7% of patients declared moderate and severe pain, 40% declared mild pain, and there were also people without any pain [15]. In total 7.9% of these patients were given opioid analgesics [15]. Similar study of McLean et al. which analyzed the medical history of 14.5 million patients transported by EMS to ED showed that 20% of patients reported moderate and severe acute pain and 17% of them obtained opioids [22]. Galinski et al. in a study of French population detected that 48% patients declared the acute pain and 73% of them were given analgesic agents, including 39% of patients who obtained a combination of at least two drugs [9]. Interesting results are shown also by Scharonow et al., where the specially trained paramedics gave their patients with acute pain morphine or fentanyl which resulted in decreasing the symptoms from the mean 7.9 to 3.3 points in the NRS at the hospital admission. Such treatment was applied in 1.8% of cases [23]. It is somewhat striking in the lights of our results where the population of patients reporting severe/extreme pain is relatively large. Interestingly, patients who did not receive pain medication and those who did received had similar scores on the second pain assessment. Due to the fact that a significant number of EMS patients are patients with trauma, it may be that the use of nonpharmacological procedures has reduced the pain or not all activities are recorded in the medical documentation by paramedics.

We have shown that the application of specific analgesic agents depends on the pain severity and ICD-compliant diagnoses. Nonopioid drugs were used more frequently. Most common states and disorders (code S00-T98), independently on the pain severity, were treated with fentanyl (about 62–70% of cases), morphine, and ketoprofen (30% each). Regarding disorders with R00-R99 code metamizole was used in 58–65% of cases and paracetamol in 54–61% of patients. The third in frequency order ICD-compliant codes I00-I99 were related to the administration of morphine (most often–18–20% of cases) and considerably more rarely ibuprofen (0–12.2%) and paracetamol (4.1–7.0%). It is not surprising that morphine is more often used in patients with cardiac diseases. As expected, opioids were prescribed more often in patients with more severe pain in all groups. These observations follow good pain management practices. However, the quite frequent use of ketoprofen is noteworthy.

The results indicate that paramedics actually realize the recommendations called “Good Practices of Pain Treatment” in most cases of application of analgesic drugs in patients. Analgesic agents are used in pain treatment regardless of its intensity appropriately according to recommendations: metamizole, paracetamol, ketoprofen, ibuprofen, and morphine in nontraumatic pain and fentanyl and morphine in pain causes by injury [24].

Multimodal analgesia was used relatively rarely (1.5–8.4% of cases), most often in patients with severe pain. Cases with more than two drugs practically did not occur in any group of patients. The synergistic effect of drugs and the reduction of their dose is important. The most common drug combinations were fentanyl with metamizole. It is in line with the practice of combining analgesics from two or more drug classes.

According to “good practices,” paramedics should reduce the pain severity by at least 50% [24]. Besides, decrease of the pain level by 50% is very vague because it will depend on the baseline pain score and status of individual patient. In case of extreme pain, reduction from 10 to 5 points is certainly relevant. On the other hand, decrease from 2 to 1 points might not mean anything from the practical point of view and not be significant for the patient. A 50% reduction of pain in any case may not be justified.

Interestingly, patients with mild pain who were not given drugs declared complete pain resolution in 6.9% of cases which is not true for patients who obtained pharmacological treatment. In the groups of patients with moderate and severe pain, the authors did not report cases of complete relief of pain regardless of application of drugs. Interestingly, both patients without drug treatment and those who were given analgesic agents show similar distribution of pain score in the first and repeated pain severity assessment. This indicates that apart from the pharmacotherapy, it is also very important to use other medical procedures. It is possible that some instrumented techniques were used to alleviate the pain instead of drugs, for example Kramer's splint in limb fractures, triangular bandage, placing the patient in a comfortable position or cold pads in case of burns. The similar phenomenon was observed in Sweden where 80% of patients declared a reduction of pain severity without getting any pain medications or obtaining any injury protection procedures [25]. One of the reasons might be that a large group of patients pays attention mostly to psychological care of medical staff. The decrease in documented pain severity could be some kind of protective documentation performed by paramedics to justify why not used pain medication. More detailed analysis whether such situations occur also in EMS in Poland is needed.

## 5. Conclusions

Our study shows that the enhancement of paramedic competences, publication of “Good practices of pain treatment,” and implementation of digital medical documentation (forcing paramedics to use pain scales in practice) had an impact on pain management by EMS. In our opinion especially introduction of obligatory pain assessment and documentation in the digital form contributed to the frequent use of pain rating scales. Unfortunately, the frequency of pain treatment is still low. We support the idea that education courses for paramedics covering pain assessment and treatment should be planned and implemented in practice. The changes in legal acts may not be enough.

### 5.1. Limitations

Our study has some serious limitations. The method of access to data contained in the emergency medical command support system (electronic documentation) in Poland and the form of these data often make it completely impossible to obtain detailed information about the patient's condition, treatment, or pain assessment scale. The timing of the second pain assessment is also problematic. Despite the fact that it should take into account the pharmacokinetics of drugs, it is often selected subjectively by paramedics. We also have no information why a pain assessment was not performed or what scale was used. There is also no certain method of assessing whether the change in pain intensity depended on the implemented medical procedures in a group of patients who did not receive pain medications.

## Figures and Tables

**Table 1:** The pain intensity, its etiology, location, and drugs recommended for the treatment.

Pain intensity	Nontraumatic pain			Traumatic pain
(NRS)	Headache	Chest pain	Abdominal pain	Trauma, burns
Mild pain, 1–4 points	Ibuprofen and/or paracetamol	Metamizole	Metamizole and/or drotaverine	Fentanyl or morphine + nonpharmacological treatment
	Paracetamol			
Moderate pain, 5–7 points	Ibuprofen and/or metamizole and/or ketoprofen	Morphine and/or metamizole	Metamizole + drotaverine	
	Fentanyl			
Severe/extreme, pain 8–10 points	Fentanyl	Morphine and/or metamizole	Morphine or fentanyl	

The table does not contain doses. Modified on the basis of good practices of pain treatment by emergency medical services.

**Table 2:** Multimodal analgesia in patients with mild, moderate, and severe pain.

		Mild pain, *N* = 53		Moderate pain, *N* = 126		Severe pain, *N* = 205	
		*n*	% (95% CI)	*n*	% (95% CI)	*n*	% (95% CI)
With nonopioid drugs	Ibuprofen-paracetamol	1	1.9 (0–5.6)	0	0	0	0
	Ketoprofen-ibuprofen	2	3.8 (0–8.9)	0	0	0	0
	Ketoprofen-paracetamol	3	5.7 (0–11.9)	13	10.3 (5.0–15.6)	10	4.9 (1.9–7.9)
	Metamizole-ibuprofen	1	1.9 (0–5.6)	0	0	0	0
	Metamizole-ketoprofen	11	20.8 (9.9–31.7)	60	47.6 (38.9–56.3)	0	0
	Metamizole-paracetamol	21	39.6 (26.4–52.8)	53	42.1 (33.5–50.7)	0	0

With opioid drugs	Fentanyl-ketoprofen	3	5.7 (0–11.9)	0	0	13	6.3 (3.0–9.6)
	Fentanyl-metamizole	6	11.3 (2.8–19.8)	0	0	62	30.2 (23.9–36.5)
	Fentanyl-paracetamol	0	0	0	0	28	13.7 (9.0–18.4)
	Morphine-ibuprofen	0	0	0	0	1	0.5 (0–1.5)
	Morphine-ketoprofen	1	1.9 (0–5.6)	0	0	14	6.8 (3.4–10.2)
	Morphine-metamizole	3	5.7 (0–11.9)	0	0	43	21.0 (15.4–26.6)
	Morphine-paracetamol	1	1.9 (0–5.6)	0	0	23	11.2 (6.9–15.5)
	Morphine-fentanyl	0	0	0	0	11	5.4 (2.3–8.5)

## Data Availability

The datasets used and/or analyzed during the current study are available from the corresponding author on reasonable request.

## Supplementary Materials

Supplementary Materials Detailed information on the size of the study groups, drugs used, and pain intensity are presented in the supplementary materials listed as follows: 1. Table S1. International Classification of Diseases (ICD-10). 2. Table S2. Pain in patients with mild pain who have not received and have received pain medications. First and second assessments. 3. Table S3. Diagnoses in patients with mild, moderate, and severe pain who have not received pain medication. 4. Table S4. Distribution of drugs in response to pain intensity in patients with mild pain 5. Table S5. Frequency of use of the drug depending on the diagnosis in patients with mild pain. 6. Table S6. Pain in patients with moderate pain who have not received and have received pain medications. First and second assessments. 7. Table S7. Distribution of drugs in response to pain intensity in patients with moderate pain. 8. Table S8. Frequency of use of the drug depending on the diagnosis in patients with moderate pain. 9. Table S9. Pain in patients with severe pain who have not received and have received pain medications. First and second assessments. 10. Table S10. Distribution of drugs in response to pain intensity in patients with severe pain. 11. Table S11. Frequency of use of the drug depending on the diagnosis in patients with severe pain.
