# Supplementary material for: Polish Experiences of Pain Treatment by Paramedics in relation to Good Practices of Pain Treatment: A Register-Based Study
Source: Emerg Med Int. 2022 Apr 7;2022:3677688. doi: 10.1155/2022/3677688 (PMC9010201; doi:10.1155/2022/3677688)
Supplement: Supplementary Materials — Detailed information on the size of the study groups, drugs used, and pain intensity are presented in the supplementary materials listed as follows: 1. Table S1. International Classification of Diseases (ICD-10). 2. Table S2. Pain in patients with mild pain who have not received and have received pain medications. First and second assessments. 3. Table S3. Diagnoses in patients with mild, moderate, and severe pain who have not received pain medication. 4. Table S4. Distribution of drugs in response to pain intensity in patients with mild pain 5. Table S5. Frequency of use of the drug depending on the diagnosis in patients with mild pain. 6. Table S6. Pain in patients with moderate pain who have not received and have received pain medications. First and second assessments. 7. Table S7. Distribution of drugs in response to pain intensity in patients with moderate pain. 8. Table S8. Frequency of use of the drug depending on the diagnosis in patients with moderate pain. 9. Table S9. Pain in patients with severe pain who have not received and have received pain medications. First and second assessments. 10. Table S10. Distribution of drugs in response to pain intensity in patients with severe pain. 11. Table S11. Frequency of use of the drug depending on the diagnosis in patients with severe pain. [file 3677688.f1.zip › 3677688.f1/ADDITIONAL_FILE_3.docx]

Table S3. Diagnoses in patients with mild, moderate and severe pain who have not received pain medication

|  | | Without drugs % (CI 95%) | | |
| --- | --- | --- | --- | --- |
|  |  | Mild pain | Moderate pain | Severe pain |
|  |  | n=174644 | n=115960 | n=48970 |
| ICD-10 code | A00-B99 | 0.2 (0.2-0.2) | 0.1 (0.1-0.1) | 0.1 (0.1-0.1) |
|  | C00-D48 | 1.1 (1.0-1.1) | 0.9 (0.8-1.0) | 1.4 (1.3-1.5) |
|  | D50-D89 | 0.1 (0.1-0.1) | 0 | 0 |
|  | E00-E90 | 1.8 (1.7-1.9) | 0.3 (0.3-0.3) | 0.2 (0.2-0.2) |
|  | F00-F99 | 2.7 (2.6-2.8) | 0.5 (0.5-0.5) | 0.2 (0.2-0.2) |
|  | G00-G99 | 1.1 (1.0-1.1) | 1.4 (1.3-1.5) | 1.6 (1.5-1.7) |
|  | H00-H59 | 0.1 (0.1-0.1) | 0 | 0 |
|  | H60-H95 | 0.3 (0.3-0.3) | 0.1 (0.1-0.1) | 0 |
|  | I00-I99 | 13.9 (13.7-14.1) | 9.3 (9.1-9.5) | 7.4 (7.2-7.6) |
|  | J00-J99 | 1.2 (1.1-1.3) | 0.6 (0.6-0.6) | 0.4 (0.3-0.5) |
|  | K00-K93 | 2.2 (2.1-2.3) | 1.9 (1.8-2.0) | 1.9 (1.8-2.0) |
|  | L00-L99 | 0.2 (0.2-0.2) | 0.1 (0.1-0.1) | 0.1 (0.1-0.1) |
|  | M00-M99 | 2.0 (1.9-2.1) | 5.4 (5.3-5.5) | 6.8 (6.6-7.0) |
|  | N00-N99 | 0.8 (0.8-0.8) | 3.4 (3.3-3.5) | 6.7 (6.5-6.9) |
|  | O00-O99 | 0.2 (0.2-0.2) | 0.7 (0.7-0.7) | 0.7 (0.6-0.8) |
|  | P00-P96 | 0 | 0 | 0 |
|  | Q00-Q99 | 0 | 0 | 0 |
|  | R00-R99 | 39.9 (39.7-40.1) | 46.9 (46.6-47.2) | 43.7 (43.3-44.1) |
|  | S00-T98 | 21.6 (21.4-21.8) | 23.8 (23.6-24.0) | 25.2 (24.8-25.6) |
|  | U00-U99 | 0 | 0 | 0 |
|  | V01-Y98 | 8.9 (8.8-9.0) | 3.6 (3.5-3.7) | 3.1 (2.9-3.3) |
|  | Z00-Z99 | 1.9 (1.8-2.0) | 0.9 (0.8-1.0) | 0.6 (0.5-0.7) |
